# Double-Dosing and Other Dangers with Non-Prescription Medicines: Pharmacists’ Views and Experiences

**DOI:** 10.3390/pharmacy6030059

**Published:** 2018-07-02

**Authors:** Natalie Gauld, Tracey Sullivan

**Affiliations:** 1 Natalie Gauld Ltd., P. O. Box 9349, Newmarket, Auckland 1149, New Zealand; 2 Independent Researcher, Wellington, New Zealand; traceyinwales@yahoo.com

**Keywords:** community pharmacy, non-prescription drugs, reclassification, self-medication, pharmacist intervention

## Abstract

The aim of this paper was to explore pharmacists’ views on reclassifications from pharmacy-only to general sales and their experiences with the supply of these medicines, in addition to pharmacists’ views on the reclassification of the shingles vaccine and sildenafil to be available through ‘accredited’ pharmacists. New Zealand community pharmacists were surveyed in 2013 with a written questionnaire of six Likert-style or open-ended questions sent to Pharmacy Guild member pharmacies. The analysis involved descriptive statistics. Responses were received from 246 pharmacies. Two thirds of pharmacists supported the reclassification of the shingles vaccine and sildenafil, although 14% disagreed with the sildenafil reclassification. Over 90% of pharmacists disagreed with the reclassification of paracetamol and ibuprofen liquids, omeprazole, naproxen, and oxymetazoline from pharmacy-only medicine to general sales. This opinion was strongest for omeprazole. With liquid paracetamol and ibuprofen, pharmacists described consumer confusion with dosing, and particularly potentially doubling-up on liquid analgesics/antipyretics including using both prescription and non-prescription variants. Many reported giving safety advice frequently. Anti-inflammatories and omeprazole were also subject to potential double-dosing, as well as requests by consumers with contraindications, precautions, and drug interactions, and for inappropriate indications. Pharmacists described various interventions, including some that were potentially life-saving. Pharmacy availability of medicines provides the potential for intervention that would not happen in a general sales environment.

## 1. Introduction

Research on the reclassification of medicines typically concentrates on the prescription to non-prescription supply. In some countries with multiple non-prescription categories, reclassification also includes pharmacy-only to non-pharmacy (general sales) supply, e.g., in the United Kingdom (UK), Canada, Sweden, Australia, and New Zealand (NZ), but this change receives little attention.

Community pharmacists are the health professionals most affected by medicine reclassification from prescription to non-prescription in most countries. They manage or oversee the management of the supply of these medicines in pharmacies. They will have insights into supplies through pharmacy and perspectives on the risks and benefits that could provide useful information to committees considering these reclassifications. However, pharmacist surveys for their opinions on reclassifications are usually for prescription to non-prescription reclassifications [1,2,3,4] and not for pharmacy-only to general sales.

For pharmacy-only to general sales reclassifications, community pharmacists will have a unique and useful perspective on the challenges consumers have with these medicines, based on their consultations. Thus, understanding the views and experiences of pharmacists on this topic may help inform the consideration of such reclassifications. However, we are unaware of any research on pharmacists’ perceptions relevant to pharmacy-only to general sales reclassifications. There is little research in NZ about pharmacists’ interventions in non-prescription supplies, or about the most serious interventions for particular categories of medicines. Research into drug-related problems in pharmacies typically provides useful information about frequencies and general nature, but does not provide details of the interventions [5,6,7]. Research into consumer behaviour shows some difficulties with the dosing of liquid analgesics [8], and some potential for double-dosing [9,10] and off-label use [10,11]. Double-dosing can involve the concomitant use of multiple products containing the same ingredient, potentially leading to double-dosing of the same ingredient, or it can involve the concomitant use of multiple ingredients in the same class, e.g., anti-inflammatories. Off-label use reflects use outside of the label conditions, e.g., indication, duration, dosing.

New Zealand has experienced a very active period of reclassification since about 2004 [12]. It has multiple non-prescription classifications: pharmacist-only medicine (a pharmacist must be involved in supply, the medicine cannot be available for self-selection, and the purchaser’s details are recorded); pharmacy-only medicine (sold from a pharmacy); and general sales medicine (available in any outlet). Some medicines have become available only through pharmacists who have undergone additional specified training, e.g., the emergency contraceptive pill, influenza vaccinations, and trimethoprim for cystitis [12]. The Medicines Classification Committee considers reclassifications and makes a recommendation to the Minister of Health’s Delegate, who makes the final decision. Following this, the agenda is published and public submissions are welcomed.

In 2013, one Medicines Classification Committee meeting agenda included two proposed prescription to non-prescription progressive reclassifications. Progressive reclassifications occur where there is a significant clinical advantage over existing non-prescription supplies; for example, a new therapeutic area, or significantly greater efficacy, or safety, or administration advantages over the existing non-prescription supplies [13]. In this case, the progressive reclassifications were for sildenafil, for erectile dysfunction, and the shingles vaccine. This meeting agenda also included multiple proposals for reclassifications of medicines from pharmacy-only to general sales categories: paracetamol liquid, ibuprofen liquid, omeprazole, topical mepyramine, oral naproxen, and nasal oxymetazoline. This study aimed to understand community pharmacists’ views about these reclassifications, and their experiences and specific considerations regarding the non-prescription provision of liquid paracetamol, liquid ibuprofen, oral non-steroidal anti-inflammatories (NSAIDs), and omeprazole. The survey results were intended for use in feedback to the Medicines Classification Committee for their consideration.

## 2. Materials and Methods

Ethics approval was not required for this survey of pharmacists, as per the Health and Disability Ethics Committee requirements in New Zealand. No pharmacists’ names were collected, nor were sensitive questions asked, nor was personal health information collected.

All member pharmacies of the Pharmacy Guild of New Zealand (approximately 700 at this time) were faxed a questionnaire by the Pharmacy Guild in September 2013. The pharmacy staff were asked to complete it and fax it back by a set time, 30 h after being sent. This timeline was short to allow collation and reporting for the Medicines Classification Committee meeting. There was no incentive to complete it, and no follow-up for non-responders.

The questionnaire was developed by the two co-authors, and checked by multiple pharmacists for face validity. It included six questions, some of which had multiple parts. One Likert-type question asked for opinions regarding each of the proposed reclassifications, with the following options: strongly agree, agree, neutral, disagree, and strongly disagree, similar to previous research [1,14]. Pharmacists’ opinions were sought for the pharmacy-only to general sales proposals for paracetamol liquid, ibuprofen liquid, omeprazole, topical mepyramine, oral naproxen, and nasal oxymetazoline. These questions were also asked for sildenafil availability through accredited pharmacists (with mandatory training), and the shingles vaccination through pharmacists trained in vaccination. Further questions were asked regarding the pharmacy-only to general sales proposals ascertaining further information about known or suspected concerns of use of paracetamol and ibuprofen liquid. These questions were based on the literature [8,9,15,16], the community pharmacy practice of the two authors of this paper, and a previous focus group of pharmacists regarding paracetamol liquid conducted by one author (unpublished). For paracetamol and ibuprofen, questions were asked about under-dosing at each dose, over-dosing at each dose, confusion about different strengths, inadvertent doubling up on the same ingredients in different products (double-dosing), more frequent use than recommended, use to help a child sleep, other off-licence use, or other problems. Pharmacists were asked to provide examples of difficulties found or their role in addressing such difficulties. For naproxen and omeprazole, an open-ended question was asked: What, if any, difficulties have you circumvented with over-the-counter (OTC) naproxen or other NSAIDs/omeprazole in your pharmacy? An opportunity was provided for any other comments. No details about the respondents were collected.

Data were entered into an excel spreadsheet, and frequencies were provided. Chi-square was used for comparisons between answers for ibuprofen and paracetamol.

Answers to open questions were analysed thematically and quantitatively, with both authors agreeing on the themes seen.

To evaluate the qualitative data, we read and reread the comments on the difficulties that pharmacists had found with these medicines or their role in addressing these difficulties. We made notes on common or recurring themes and selected comments that most reflected the concerns pharmacists had over the proposed reclassifications.

## 3. Results

Two hundred and forty-six responses were received, providing answers from over one third of the pharmacies faxed.

### 3.1. Views on the Reclassification of the Shingles Vaccine and Sildenafil

**Figure 1:**
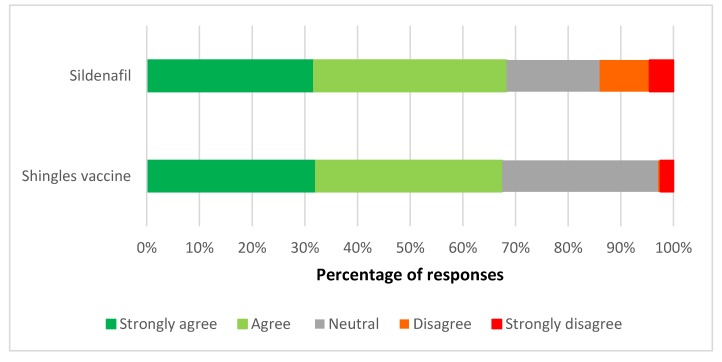
Views on the reclassification of the shingles vaccine and sildenafil to allow supply by accredited pharmacists.

Respondents were supportive of the reclassification of both the shingles vaccine and sildenafil to allow supply by pharmacists with additional mandated training/accreditation for supply (Figure 1). Two thirds (66.3%) of pharmacists agreed or strongly agreed with the shingles vaccine reclassification, and 67.5% with the sildenafil reclassification. A small but important minority of pharmacists disagreed or strongly disagreed with sildenafil being reclassified (13.8%), while only 2.8% disagreed with reclassifying the shingles vaccine. There was no response to this question from three respondents each.

### 3.2. Views on the Reclassification of Paracetamol and Ibuprofen Liquids to General Sales

**Figure 2:**
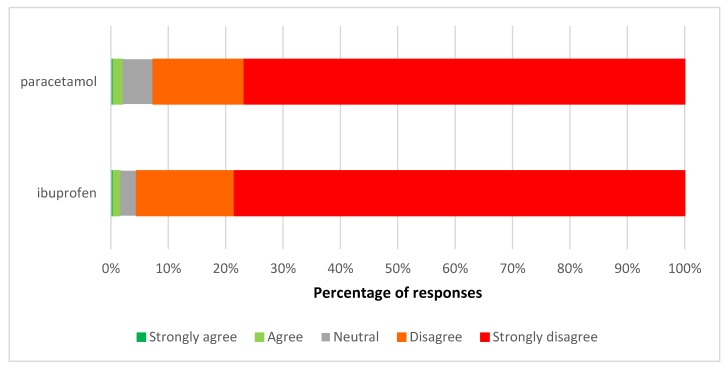
Views on the proposed pharmacy-only to general sales reclassification of paracetamol liquid and ibuprofen liquid.

Few respondents agreed with the proposal for ibuprofen liquid or paracetamol liquid to become available outside of pharmacies. Most respondents (78.5% for ibuprofen and 76.7% for paracetamol) strongly disagreed with this proposal (Figure 2).

**Figure 3:**
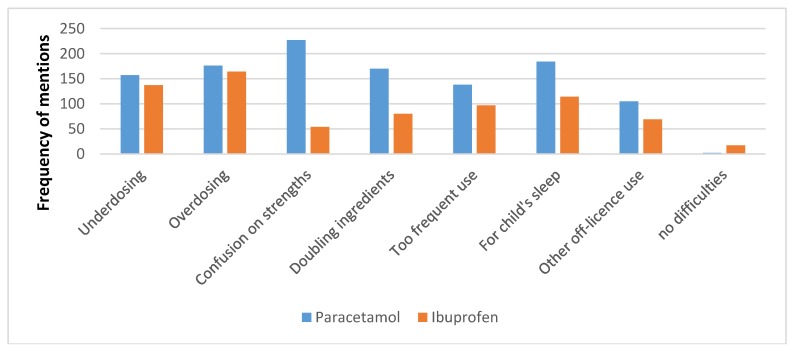
The frequency of respondents reporting concerns they experienced with purchasers of paracetamol or ibuprofen liquids.

The reasons for respondents’ concern are presented in Figure 3. The most common concern was confusion about strengths of the paracetamol liquid (available in 120 mg/5 mL and 250 mg/5 mL), reported by 92.3% of respondents. Under-dosing and over-dosing were reported by over half of the respondents in both the ibuprofen and paracetamol groups. Too frequent use was reported by 56.3% of respondents about paracetamol and 40.1% about ibuprofen (Chi-square = 13.26, *p* = 0.0027). Two respondents (0.8%) reported no difficulties with paracetamol liquid, and 17 (6.9%) with ibuprofen.

### 3.3. Experiences and Advice with Paracetamol and Ibuprofen Liquids

When invited to provide examples of the difficulties found or their role in managing difficulties, most respondents did so. There was a concerning and overwhelming picture of pharmacists finding confusion amongst parents and caregivers around the different products, doses, and strengths, resulting in actual or intended inappropriate practices in which pharmacists or pharmacy staff intervened. Comments indicated that some purchasers lacked understanding about safety aspects of these medicines, including double-dosing with prescribed and non-prescription paracetamol or ibuprofen, or not recognizing that multiple medicines contain paracetamol liquid or ibuprofen (e.g., ibuprofen from the doctor and Nurofen bought from the pharmacy). The following quotes selected by the authors of the paper are representative of many such statements from pharmacists:

“Parents don’t often know the correct amount of paracetamol to give to their kids and different strengths of paracetamol make it even more confusing.”

“People not realising different brands [are] same thing (Panadol, Pamol), people not realising how dangerous paracetamol is in overdose, considered “safe” cos it’s common.”

“Some parents dose paracetamol around the clock e.g., q4h = six doses in 24 h. Many parents think orange flavour is stronger, strawberry is weaker, but depends on brand!”

“Double-dosing for more pain relief, dosing more frequently even with well-educated patients.”

Some comments mentioned demand for use in young babies for whom the medicines were not appropriate without medical advice, particularly for ibuprofen.

“Using as first line pain relief for very young babies—Nurofen has been aggressively marketed to parents of young babies.”

A small number of concerns for ibuprofen or paracetamol related to parents’ desires to delay or avoid seeking medical advice, e.g., not going to the doctor when it did not work, or using for possible rheumatic fever, with a burst ear drum, or at night instead of seeing the doctor. Pharmacists were concerned that there would be no health professional to intervene in these instances should it become available outside of pharmacies. With ibuprofen, some noted having to advise against planned use by parents when a child is not eating or drinking or has vomiting or diarrhoea. Furthermore, one mentioned planned use in a child with possible kidney disease, and others reported use in children with asthma.

Respondents often indicated actions taken, typically dose clarification, supply of a dosing chart, appropriate measurements of doses, and weighing children to ensure that the dosing was correct. They commonly mentioned discussing the appropriate indication for use and advising against dosing with other medicines containing the same ingredient (e.g., prescribed paracetamol or ibuprofen),

“Very common for parents to not update dose according to weight. We try to advise [at] every visit/purchase.”

“Giving information on correct dose and frequency. Pointing out that it will only help child to sleep if they have pain or fever relieved by the paracetamol.”

“We remind people … not to double-up. Have had incidences of wrong doses, doubling-up, confusion over use, strength. We ask for children’s weight to check paracetamol and ibuprofen doses.”

Three-quarters of respondents (74.8%) were aware of cases where paracetamol was being used to help a child go to sleep, versus 46.3% for ibuprofen (Chi-square = 40.23, *p* < 0.00001, df = 1).

“People always want to use it [paracetamol] for sleep.”

Other uses outside of the licensed indications were reported for paracetamol and ibuprofen liquids. Examples included use for runny noses, cough, hayfever, and a crying infant when leaving at kindergarten, particularly for paracetamol. Multiple respondents reported people trying to buy ibuprofen liquid for stomach upset.

“Customers think that paracetamol is a cure for lots of children’s medical problems.”

“Use for cough or sleep aid. If child is grumpy may use paracetamol. When prescription given out or sold OTC, we always counsel.”

“Purchasing for vomiting + diarrhoea [ibuprofen].”

There were a couple of mentions of language barriers also creating difficulties for consumers in understanding the correct use of paracetamol or ibuprofen liquid.

### 3.4. Views on the Reclassification of Omeprazole, Naproxen, and Nasal Oxymetazoline to General Sales

**Figure 4:**
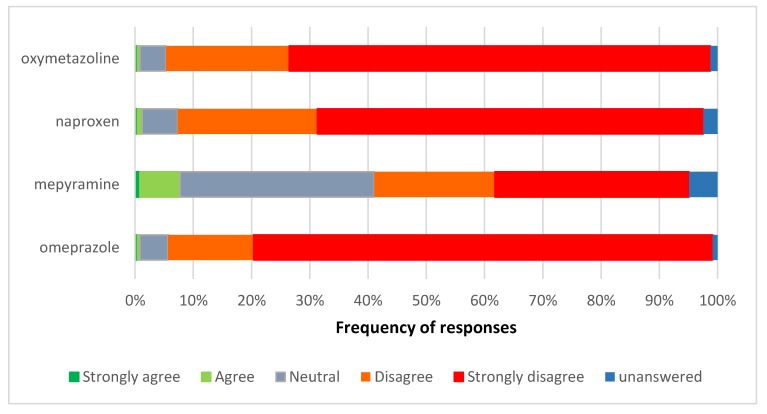
Views on the proposed pharmacy-only to general sales reclassification of omeprazole, naproxen, nasal oxymetazoline, and topical mepyramine.

There was strong disagreement with the proposals for omeprazole, naproxen, and nasal oxymetazoline to move from pharmacy-only to general sales (Figure 4). For omeprazole and oxymetazoline, 93.5% of respondents disagreed or strongly disagreed with the proposed reclassification. For naproxen this was slightly lower at 90.2%, and mepyramine was much lower at 54.1% of respondents. Respondents’ reactions were strongest for omeprazole.

### 3.5. Experiences with Omeprazole

Comments from pharmacists revealed considerable concern about the potential for missing serious and life-threatening medical conditions such as heart attacks, cancer, peptic ulcers, and gastric bleeds should omeprazole move to general sales. More than a quarter of pharmacists (*n* = 64, 26.0%) commented that they had referred patients to a doctor with the result of a serious medical diagnosis being made for these patients. A further 11.0% of pharmacists (*n* = 27) reported that they had referred patients who had described ‘red flag’ symptoms to their general practitioner (GP), but did not mention the outcomes.

Twenty-one pharmacists (8.5%) commented that they had recognised one or more patients’ symptoms as either angina or heart attack and referred them for immediate medical treatment, often with knowledge of the outcome (e.g., that a myocardial infarction (MI) or angina was diagnosed).

“Patients have problems differentiating between angina and heartburn.”

“When patient thinks it’s heartburn but actually was angina symptoms. Told to get to doctor, Nitrolingual [glyceryl trinitrate] prescribed.”

“… patient was having heart attack, treating with antacids and then came in for omeprazole but had pain in shoulders, jaw, sent to [doctor], ended up in ambulance with heart attack, came back and thanked me.”

“A customer wanted some but on discussion of symptoms sent to GP as symptoms suggestive of MI. Correct, had triple bypass surgery in subsequent month.”

“Patients presenting with cardiac symptoms but thinking it’s indigestion; patients over 50 [years of age] with indigestion not going to [doctor] and buying antacids; a couple of instances where we have had to call ambulance [for possible] heart attack.”

Seven pharmacists mentioned they had intervened on patients who had been buying omeprazole on a long-term basis or taking increased quantities and had referred them to a doctor, resulting in a cancer diagnosis.

“When questioning patient, found out had serious problem, [doctor] in UK had prescribed omeprazole but I sent him to a local doctor—turned out he had throat cancer, now on chemotherapy and had throat surgery. This would not have been picked up by selling product in supermarket.”

“Overuse—had a customer that we referred and turned out to be stomach cancer. Life saved!”

“Stopped young male patient self-medicating and had gastroscopy- gastric cancer discovered and treated.”

“Blood disorders and two cases of identifying stomach cancers.”

“Long-term epigastric pain ignored and/or self-treated; patient had cancer and died.”

It was common for pharmacists to advise patients to seek medical advice regarding symptoms indicating they may have a peptic ulcer, *Helicobacter pylori* infection, or even a gastric bleed.

“Regular patient who comes in regularly for omeprazole, we advised them to visit doctor as he was increasing dose of OTC omeprazole—eventually found out he had an *H. pylori* infection.”

“… increased use and when advised to go to the doctor, had a perforation.”

“Regular use without diagnosis from [doctor]—bleeding stomach ulcer resulting in hospital admin.”

“Had a few patients that needed to be referred to [doctor] with peptic ulcer concern.”

There was real concern from pharmacists regarding the number of times they intervened with patients who were taking interacting medicines such as warfarin, with 22 pharmacists (8.9%) noting their interventions. Some patients were prevented from doubling-up with prescription proton pump inhibitors (PPIs, mentioned by 11 pharmacists, 4.5%), while others were on long-term NSAIDS. Sixty-two pharmacists (25.2%) mentioned intervening in the overuse or long-term use of omeprazole without consulting with a doctor. Seven pharmacists (2.8%) reported finding the cause of the patient’s gastric complaints to be the result of taking NSAIDS inappropriately.

“Advertised on TV and misinterpretation of ad symptoms frequent—often [we] check history and find [that the patient is] already on an H2 antagonist or PPI.”

“People taking NSAIDS (four or more [patients]) and not consulting doctor.”

Misperceptions by the public about the use of this medicine or avoiding the doctor were also reported.

“Customers think it is appropriate to take this OTC pretty regularly. Also had a case of a lady buying it because the doctor wouldn’t prescribe it as the doctor wanted her to have a gastroscopy.”

Two pharmacies reported refusing the sale of omeprazole requested for use in a child.

“… had a customer try to buy it for 12-week-old twin babies then changed her story so that she could just purchase it, still with the intention of being able to give to her friend’s babies who were having difficulties. Sale refused on all grounds. Explained dangers and also highlighted to other local pharmacies just in case she still attempted to get [it].”

One pharmacist reported pregnant women requesting to purchase omeprazole.

### 3.6. Experiences with Naproxen and Other NSAIDs

Three respondents wrote “none” or “nil” in response to the question about naproxen or other NSAIDs. A further 11.8% did not answer this question. The remaining respondents reported difficulties averted with non-prescription naproxen or other NSAIDs in their pharmacy or noted reasons why these medicines should not become available in the supermarket. These reasons were safety focused. Throughout the comments was a common theme of a “blasé attitude”, a belief that these medicines are safe, misunderstandings, and that “patients don’t know”. Cases of inappropriate recommendation by another health professional were reported, one with a dentist in pregnancy, another with a nurse (mentioned below), and others mentioning physiotherapists’ and chiropractors’ recommendations in patients with contraindications or precautions.

Preventing double-dosing with other NSAIDs (taking another NSAID concomitantly) was mentioned by 93 respondents (43.9% of those providing comments, 37.8% of all respondents), with some indicating that it was a common problem. Often there was a suggestion that patients do not understand that the medicines are from the same class. Sometimes other non-prescription brands were mentioned in the double-dosing, as were prescription NSAIDs.

“People not realising already taking another NSAID e.g, Nurofen [non-prescription ibuprofen brand] or Voltaren [diclofenac] + Nurofen. [This] happens quite frequently.”

“Misunderstanding of clients of the difference and similarities of anti-inflammatories. It is not unusual to find patients taking [two or more].”

Some comments perhaps indicated confusion arising from the different indications for which products are known.

“Patients taking diclofenac wanting to buy naproxen for period pain, Nurofen cold and flu [ibuprofen with a decongestant], with Nuromol [paracetamol and ibuprofen].”

Pharmacists reported regularly intervening where contraindications or precautions for NSAID use were found, such as pregnancy, renal impairment, asthma, reflux, ulcers, or old age. This was mentioned in 46.7% of comments (40.2% of all respondents). Some pharmacists reported that people with chronic illness lacked awareness of the concerns of an anti-inflammatory, e.g., people with unstable hypertension or renal impairment.

“Pregnant lady taking NSAIDs for months that she had bought from a supermarket.”

“Several times a year find people with stomach ulcers want to buy NSAIDs.”

“Patients allergic to aspirin didn’t realise could be allergic to other NSAIDs. Patients with stomach ulcers wanting to buy, not realising risks.”

“Older diabetic patients trying to buy multiple boxes, same with hypertensive patients.”

“Only yesterday patient with cardiac surgery history wanted naproxen because recommended as safe by a nursing friend.”

Sometimes multiple concerns would arise for the same patients.

“On questioning patient is on blood thinning meds, had previous or currently has stomach issues (ulcers).”

“Elderly customers asking for NSAIDs when they have [gastro-intestinal] problems or cardiovascular disease or both.”

“Often this type of medication is requested by patients on many meds and [those who] have chronic pain issues. Often on PPIs.”

The potential for drug interactions was raised by 39.1% of pharmacists providing comments (33.7% of all respondents). Many reported having stopped patients from taking an NSAID while on medicines such as lithium, warfarin, blood pressure medications, or diuretics, and nine mentioned the “triple whammy” of an NSAID with a thiazide and an ACE inhibitor.

“Medicine interactions is the most common issue—many people think that NSAIDs are ok with all meds especially because they can buy Nurofen [ibuprofen] from the supermarket!”

“Triple whammy a number of times.”

Five pharmacists reported patients requesting NSAIDs for use before sport to prevent pain in case of an injury, for use in marathons, or for use within the first 48 h of injury.

“Sports people using it before sports—potential for kidney damage—refer to Panadol [paracetamol], using anti-inflammatories before 48 h after injury.”

Often, multiple concerns were raised by pharmacists.

“Elderly patient asking for NSAIDs (diclofenac), the doctor had previously stopped it 2 years ago due to side effects, but patient is unable to see why we refused sale when she ‘really needs it’. Patient taking Naproxen and wanting diclofenac. Patient wanting NSAIDs at first injury (i.e., within 48 h). Patient wanting NSAIDs and hasn’t trialed any other type of pain relief. Asthmatic patient wanting NSAIDs.”

Some respondents suggested that patients had a lack of awareness about side effects or precautions for use. In 11.8% of comments (10.2% of all respondents), the pharmacist raised concerns about inappropriate dosing.

“Blasé attitude about this often and how much to give. People believe it is very safe to use, anywhere, anytime.”

“Continually using NSAIDs causing stomach bleeds, advised alternative pain relief.”

Inappropriate indications for use arose, e.g., sore stomach and pain from stomach ulcers. Seven pharmacists mentioned requests of non-prescription anti-inflammatories for gout.

“Being used to treat gout but patient not on preventive medication—sent to [doctor], explained need to lower uric acid levels.”

## 4. Discussion

This research provides insight into the concerns seen in pharmacies with patient requests for selected non-prescription medicines, and the interventions made. The research was unusual in seeking opinions on pharmacy-only to general sales reclassifications, as well as two prescription to non-prescription reclassifications of medicines that had not been reclassified elsewhere at that time. The findings provide useful information for health policy considerations around access to medicines.

Respondents were largely positive about the reclassification of the shingles vaccine and sildenafil. We are not aware of other peer-reviewed literature on pharmacists’ opinions on a sildenafil reclassification for comparison. Complex reclassifications have not always been received positively. For example, pharmacists were less supportive of the UK simvastatin reclassification [17]. Possibly pharmacists see sildenafil differently compared to simvastatin; for example, some pharmacists expressed doubts about the efficacy of the 10 mg reclassified simvastatin strength. In NZ, reclassification to availability through ‘accredited’ pharmacists, as has occurred for vaccinations, the emergency contraceptive, and trimethoprim, may increase confidence. Confidence may also have grown with the experience of other complex reclassifications in NZ (e.g., oseltamivir, sumatriptan, trimethoprim, and other vaccinations), and the training and tools for the supply that were usually used with these medications [12]. Trimethoprim and oseltamivir reclassifications were well-regarded by pharmacists [18,19]. Allowing only accredited pharmacists to supply these medicines may also satisfy pharmacists who do not want to provide the service but are happy for others to do so. Both the shingles vaccine and sildenafil have since been reclassified [13].

Most respondents strongly disagreed with the proposed general sales reclassifications of paracetamol liquid, ibuprofen liquid, oxymetazoline, naproxen, and omeprazole, with multiple concerns about patient safety raised for paracetamol, ibuprofen, naproxen, and omeprazole. Their reactions were stronger about a move to general sales than their reactions to the sildenafil and shingles vaccine reclassification had been. The mepyramine reclassification to general sales was less strongly opposed. Many respondents were likely to be pharmacy owners and could have vested interests in keeping medicines available in the pharmacy, but their comments often indicated serious concerns. The Pharmacy Guild provided these survey responses within their submission to the Medicines Classification Committee prior to their consideration of the reclassifications. While topical mepyramine and oxymetazoline have since been reclassified in NZ to general sales, the other medicines remain pharmacy-only.

For paracetamol liquid, pharmacists found consumer confusion between different brands, strengths, and flavours of the liquid formulations. In addition, dosing children by age rather than weight is commonly reported. Dosing and usage outside the recommended indications for paracetamol and ibuprofen appeared to be commonly encountered and counselled against in pharmacies. An Australasian study found most parents of young children understood the frequency of dosing correctly for paracetamol (93%), and somewhat less for ibuprofen (67%), but only 51% calculated a correct weight-based dose [8]. An Israeli study found that parents often used excessive dosing or repeated doses too frequently when giving antipyretics to children [16]. The NZ Poisons Centre lists paracetamol as the most common substance involved in the poisoning of children under the age of 5 years, and concerns about paracetamol make up 60–65% of calls to the Centre [20]. Given the widespread confusion over brands/strength/dose reported in this study with prescription and non-prescription supplies (65% of pharmacies mentioned this), perhaps an extra consulting fee may have merit to provide comprehensive counselling and dosing aids for liquid paracetamol and ibuprofen for every prescription supply.

Most pharmacists described advice provided with paracetamol and ibuprofen liquids. Some pharmacists described intervention on either every prescription or OTC sale to ensure that parents and caregivers were dosing their children according to weight, rather than approximate age. These could help avoid both sub-therapeutic dosing (and potentially a doctor’s visit for unresolved pain or fever) and overdosing, which can cause serious concerns. It was rare for respondents to note they had no difficulties with paracetamol or ibuprofen. Few respondents did not describe interventions on paracetamol or ibuprofen. It is possible that a small number do not provide this information, perhaps having very few paediatric patients or believing that consumer knowledge is greater than it is. Also, some pharmacists may have chosen to answer only the tick box questions on the survey given the short timeframe. However, a Swedish mystery shopping study found that 56% of requests in a pharmacy for the treatment of a child with fever gave no advice [21]. Further research examining the frequency of and safety benefits of counselling about ibuprofen and paracetamol liquids in pharmacy is warranted.

Pharmacists described some important and potentially life-saving interventions with omeprazole, naproxen, and other NSAIDs. In particular, there seemed to be a lack of awareness of some consumers to potential dangers. Such dangers include concomitant medical conditions, interactions, and the dangers of double-dosing with other medicines containing the same ingredient or the same class of ingredient. Some interventions described would have reduced the risk of an adverse event, e.g., double-dosing, and use with interactions or contraindications or precautions. Other interventions were about an underlying condition needing urgent attention or needing to be ruled out. Non-prescription consultations about or requests for pain or gastrointestinal drugs have been associated with a greater likelihood of pharmacists finding drug-related problems than in many other conditions [5,6]. A United States (US) study found inadequate knowledge about analgesics in consumers, including some with precautions for use, but after receiving some education, four out of five consumers said they would change their behaviour [22]. Another US study found that consumers without medical training generally did not understand the risks of double-dosing with different medicines containing the same ingredient, but could respond to education [9]. An NZ study conducted in the 1990s found that 1.6% of people taking diclofenac tablets reported the use of a prescription NSAID on the same day, and 2.4% reported the use of another non-prescription NSAID on the same day [10] However, it is not clear if there was a switch through the day between one NSAID and another versus doubling dosages. It is reasonable to expect that double-dosing would increase without intervention in the pharmacy if the medicines became available through general sales, thus placing people at risk.

Greater consumer education about non-prescription medicines appears warranted. Perhaps doctors and pharmacists need to regularly advise patients that their medical condition or medication they are taking may affect other medicines they could buy without a prescription and to always check with a doctor or pharmacist before taking non-prescription medicines. This seems particularly important when people may purchase some medicines from the supermarket, or use medicines in the home.

This research informed a Medicines Classification Committee consideration of prescription to non-prescription reclassifications, and pharmacy-only to general sales reclassifications, with most considerations aligning with the pharmacists’ recommendations. 

### Strengths and Limitations

The response rate of the questionnaire was reasonable, particularly given the timeframe. Around a quarter of NZ pharmacies were not members of the Pharmacy Guild and therefore were not surveyed. It is possible that they could have held different opinions.

We have provided a large range of quotes in this paper to be as informative as possible to pharmacists, pharmacy technicians, and medicines counter assistants, to help them reflect on their current practice, and to students and pharmacy interns to provide a real-life indication of the need for questioning and harms they need to prevent.

We did not pilot the questionnaire owing to time restrictions. Many questions arose from the experiences of the authors in community pharmacy and reports from other pharmacists, because there has been little prior published research of pharmacists’ experiences with liquid analgesics.

Given the short turnaround for the study, and the limited space to write, some respondents may have provided some top of mind concerns rather than the full range of concerns they had found. Had we asked specific questions about risk factors they had encountered, we may have had a higher proportion of pharmacists reporting this. Some pharmacists work in pharmacies in low socioeconomic areas in which non-prescription sales are minimal, and therefore may not experience the concerns others do.

We did not receive demographics for the pharmacists to describe our sample or explore differences in answers by demographics. We also only asked about medicines that were being considered for reclassification at a particular meeting, rather than a wider range of medicines, as done in other studies [1,2]. We did not survey pharmacy assistants for their views and experiences, which may have differed from those of the pharmacists. We did not question the reasoning behind the sildenafil or shingles vaccine answers.

## 5. Conclusions

Pharmacists held safety concerns about possible pharmacy-only to general sales reclassifications of paediatric paracetamol, paediatric ibuprofen, naproxen, and omeprazole, with a wide range of interventions described for all. They were more positive about the shingles vaccine and sildenafil becoming available through ‘accredited’ pharmacists. Pharmacists’ views and experiences can be helpful in understanding the potential risks of pharmacy-only to general sales reclassifications, for committees considering reclassifications, and to raise awareness for pharmacists, other pharmacy staff, and students.
